# ANCA-Negative Microscopic Polyangiitis With Gastrointestinal and Renal Involvement: A Diagnostic and Therapeutic Challenge

**DOI:** 10.7759/cureus.86304

**Published:** 2025-06-18

**Authors:** Devon D Thorpe, Shahtaj Shah, Ekene Okeke, Nargiz Gasimova, Deepika Jain

**Affiliations:** 1 Internal Medicine, Overlook Medical Center, Summit, USA; 2 Nephrology, Overlook Medical Center, Summit, USA

**Keywords:** acute kidney injury, anca-negative vasculitis, enteritis, gastrointestinal manifestations, microscopic polyangiitis (mpa)

## Abstract

We present the case of a 64-year-old male with acute kidney injury (AKI) and gastrointestinal symptoms. Computed tomography (CT) revealed inflammatory changes in the lungs and colon, and push enteroscopy confirmed enteritis. Initially, AKI was attributed to GI losses causing prerenal azotemia, but persistent proteinuria prompted kidney biopsy, confirming anti-neutrophil cytoplasmic antibody (ANCA)-negative microscopic polyangiitis (MPA). While induction immunosuppression was planned, the patient developed necrotizing fasciitis requiring treatment deferral. Renal function ultimately normalized with steroids alone, highlighting the need for individualized therapy in seronegative vasculitis. This case underscores the diagnostic challenge of ANCA-negative MPA and the pivotal role of histopathology and multidisciplinary collaboration.

## Introduction

Microscopic polyangiitis (MPA) is a pauci-immune small-vessel vasculitis associated with anti-neutrophil cytoplasmic antibodies (ANCAs), primarily targeting myeloperoxidase (MPO) [1]. Classic presentations include rapidly progressive glomerulonephritis and alveolar hemorrhage, though multisystem involvement can occur [2,3]. Gastrointestinal (GI) manifestations, such as abdominal pain, bleeding, or enteritis, though rare, may mimic infectious or inflammatory conditions, delaying diagnosis. ANCA seronegativity, which occurs in approximately 10% of cases [3], further masks the diagnosis, necessitating reliance on histopathology. While renal biopsy typically reveals crescentic glomerulonephritis with minimal immune deposition [4], extrarenal findings (e.g., pulmonary nodules or GI inflammation) may provide additional clues [5]. The pathogenesis of ANCA-negative MPA is poorly understood, though molecular mimicry (e.g., anti-LAMP-2 antibodies triggered by bacterial antigens) has been proposed [6]. We describe a case of ANCA-negative MPA presenting with AKI, jejunal ulcerations, and pulmonary infiltrates. Initially attributed to volume depletion, the patient’s persistent proteinuria led to a renal biopsy and definitive diagnosis. This case emphasizes the importance of considering vasculitis in multisystem illness, even without serological support, and the pivotal role of tissue diagnosis [7].

## Case presentation

A 64-year-old Caucasian male with a history of hypertension controlled on amlodipine 2.5 mg daily presented to the emergency department with a five-day history of nausea, non-bloody, non-bilious vomiting, abdominal pain, and petechiae. Although he recounted intermittent cough and subjective fever weeks prior, he denied any other constitutional or coryzal symptoms, and both the preceding symptoms were absent at the time of his presentation to the emergency department. He had previously been evaluated at an urgent care center, where no significant findings were noted, and was prescribed doxycycline for suspected bacterial enteritis, which he completed prior to arrival at the hospital. He denied recent over-the-counter medication use, exposure to contrast agents, travel, or exposure to sick contacts.

On examination, his vitals were stable, with a blood pressure of 108/52 mmHg, a pulse rate of 63 beats per minute, and SpO2 of 92% on room air. The physical examination’s significant findings were a bilateral, non-tender, non-blanching purpuric rash extending from the thigh regions to the plantar aspect of both feet. There was no evidence of synovitis, inflammatory eye disease, serositis, or any other clinical manifestations of an underlying rheumatologic disease.

Initial laboratory workup (Table 1) revealed abnormal renal function with a serum creatinine of 8.19 mg/dL and a blood urea nitrogen (BUN) of 92 mg/dL.

**Table 1:** Key laboratory values on admission and at discharge The table summarizes key electrolyte, renal, autoimmune, and infectious disease markers. Notable abnormalities at presentation included hyperkalemia, azotemia, elevated phosphorus, and an elevated anion gap metabolic acidosis, all of which improved with treatment. Autoimmune serologies—including anti-MPO, anti-PR3, ANA, and anti-GBM antibodies—were negative, reducing suspicion for systemic vasculitis or lupus nephritis. Infectious serologies for hepatitis A, B, C, and HIV were also negative. Values in parentheses indicate qualitative interpretation when applicable. Anti-MPO, myeloperoxidase antibody; anti-PR3, protease 3 antibody; anti-GBM, anti-glomerular basement membrane; p-ANCA, perinuclear anti-neutrophil cytoplasmic antibodies

Test	Admission Result	Discharge Result	Normal Range
Sodium	134 mmol/L	141 mmol/L	135–145 mmol/L
Potassium	5.7 mmol/L	3.3 mmol/L	3.2–4.9 mmol/L
Chloride (Cl)	108 mmol/L	107 mmol/L	95–110 mmol/L
CO₂ (bicarbonate)	16 mmol/L	21 mmol/L	21–32 mmol/L
Blood urea nitrogen	92 mg/dL	40 mg/dL	7–18 mg/dL
Creatinine	8.19 mg/dL	2.08 mg/dL	0.6–1.3 mg/dL
Anion gap	16 mEq/L	7 mEq/L	5–15 mEq/L
Calcium	8.1 mg/dL	7.5 mg/dL	8.5–10.1 mg/dL
Phosphorus	6.9 mg/dL	3.1 mg/dL	2.5–4.8 mg/dL
Anti-MPO	3.9 U/mL (negative)	N/A	<20 U/mL
Serum anti-PR3	5.9 U/mL (negative)	N/A	<20 U/mL
Atypical p-ANCA	<1:20 (negative)	N/A	<1:20
Complement 3	112 mg/dL	N/A	90–180 mg/dL
Complement 4	23 mg/dL	N/A	10–40 mg/dL
A1c	5.3%	N/A	4.0–5.6%
Antinuclear antibody	Negative	N/A	Negative
Rheumatoid factor	Negative	N/A	Negative
Cryoglobulins	Negative	N/A	Negative
Anti-GBM antibody	Negative	N/A	Negative
HAV antibody	Negative	N/A	Negative
HBV surface antigen	Negative	N/A	Negative
HCV antibody	Negative	N/A	Negative
HIV antigen/antibody	Negative	N/A	Negative

Urine studies revealed microscopic hematuria and acute tubular necrosis assessed at that time to be caused by prerenal azotemia. Protein-to-creatinine ratio was 0.39 g/g (normal range: <0.2 g/g) as depicted in Table 2.

**Table 2:** Urine investigations The three result columns demonstrate the temporal progression of proteinuria and urinary sediment findings. The observed sediment abnormalities (including pyuria and hematuria) were initially attributed to traumatic Foley catheter insertion. This dissociation between transient catheter-related changes and persistent pathological markers was crucial in guiding further diagnostic evaluation. HPF, high power field; LPF, low power field

Test	Admission Result	Post-Discharge Result	Repeat Outpatient	Normal Range
Protein-to-creatinine ratio	0.39 g/g	0.926 g/g	1.7 g/g	<0.2 g/g
WBC (per HPF)	>50	–	–	0–5
RBC (per HPF)	>50	–	–	0–5
Casts (per LPF)	0–5 (none pathological)	–	–	0–5
Bacteria	Few	–	–	None
Leukocyte esterase	Positive	–	–	Negative
Nitrates	Negative	–	–	Negative
Urine culture	No growth	–	–	No growth
Urine sodium	<20 mmol/L	–	–	20–30 mmol/L
Urine potassium	45 mmol/L	–	–	25–125 mmol/L
Urine creatinine	240 mg/dL	–	–	20–250 mg/dL

Given his predominant enteritis-type symptoms on admission, a limited inflammatory and infectious workup was performed (Table 3). A comprehensive gastrointestinal pathogen PCR panel (Appendix A) was performed, testing for 22 bacterial, viral, and parasitic targets, all of which returned negative results.

**Table 3:** Stool analysis: inflammatory, infectious, and occult blood testing *Summary of stool investigations including fecal occult blood, calprotectin, and multiplex PCR GI panel testing. GI, gastrointestinal; PCR, polymerase chain reaction

Test	Result	Reference Range/Units
GI panel (PCR test)	Negative	*
Fecal occult blood test	Positive	Negative
Fecal calprotectin	204 mcg/g	<50 mcg/g (negative)

**Figure 1:**
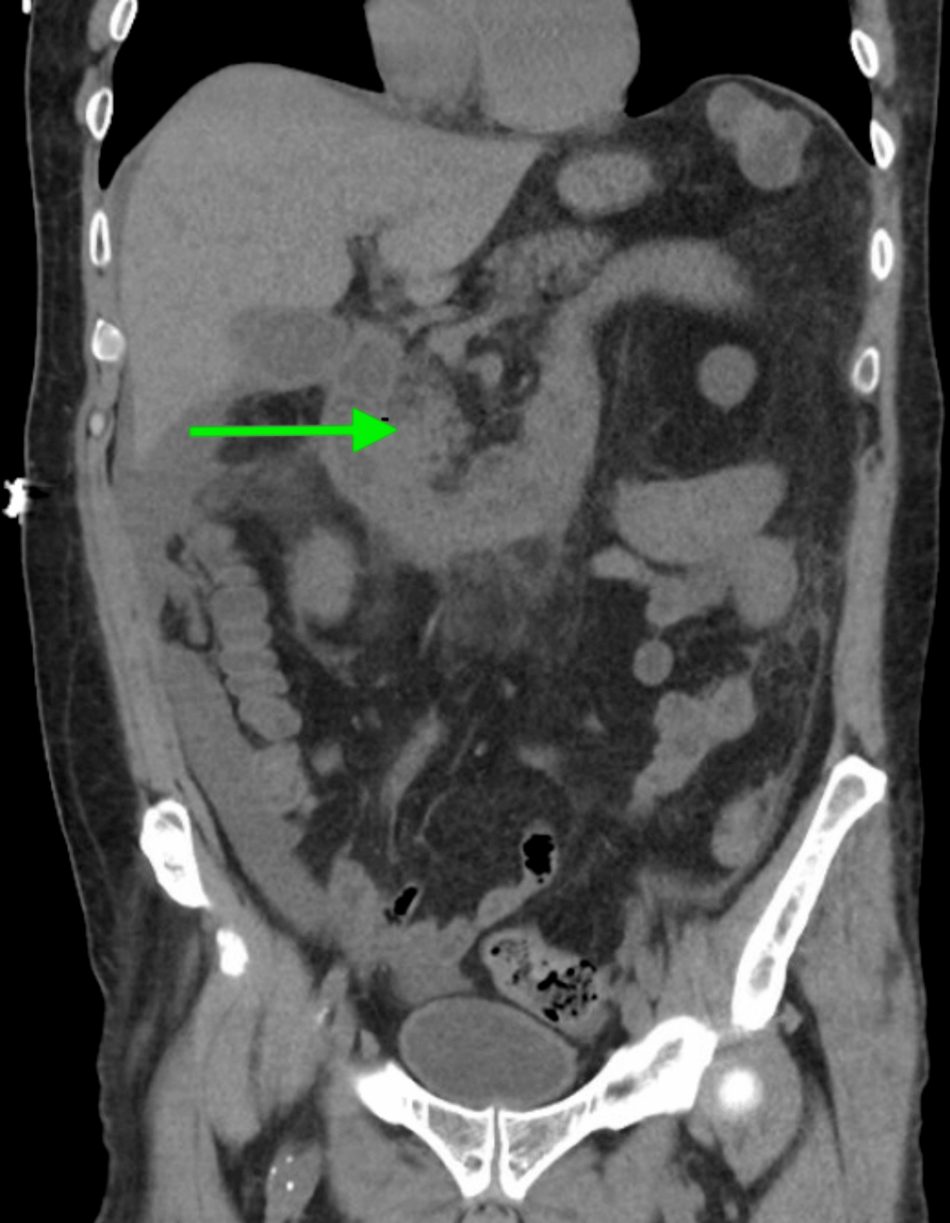
Abdominal CT (coronal view) showing diffuse small bowel wall thickening suggesting vasculitic enteritis Coronal section from a non-contrast CT of the abdomen and pelvis demonstrating diffuse small bowel wall thickening (green arrow), most prominent in the jejunum, consistent with inflammatory enteritis and suggestive of gastrointestinal involvement in microscopic polyangiitis.

Computed tomography (CT) of the abdomen and pelvis showed diffuse small bowel wall thickening, suggestive of infectious or inflammatory enteritis (Figure 1), while the CT of the chest (without contrast) revealed nodular bibasilar infiltrates and small pleural effusions, suggesting an inflammatory versus infectious etiology. Given concerns for cough and subjective fever prior to admission, a possible underlying pneumonia was entertained, prompting his completion of a week’s course of ceftriaxone inpatient.

**Figure 2:**
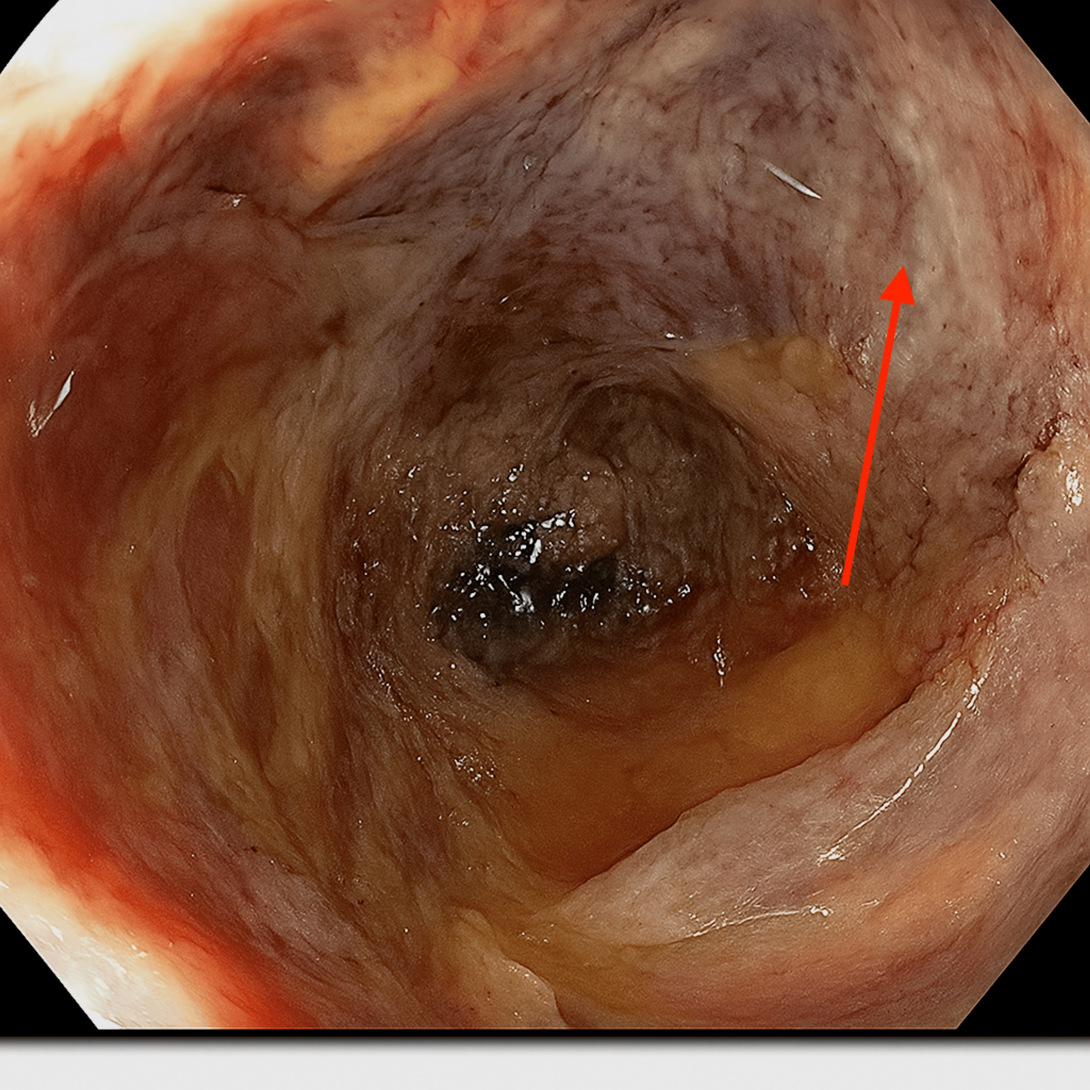
Push enteroscopy revealing a jejunal lesion Endoscopic image showing a partially circumferential, friable, non-obstructing polypoid mass in the proximal to mid jejunum (red arrow). Biopsy confirmed active enteritis with ulceration and fibroinflammatory debris, consistent with gastrointestinal involvement in microscopic polyangiitis.

The nephrology team initially attributed the acute kidney injury to prerenal azotemia from volume depletion due to GI losses. He was started on bicarbonate-containing isotonic fluids, and a Foley catheter was placed to allow monitoring of urine output. The gastroenterology team, concerned about small bowel thickening, performed a push enteroscopy, revealing a partially circumferential, friable, non-obstructing polypoid mass in the proximal to mid jejunum with satellite lesions distally. Biopsy results indicated active enteritis, which was initially presumed to be infectious while inpatient (Figure 2).

The patient’s renal function improved during his nine-day hospitalization, with creatinine decreasing to 2.08 mg/dL at discharge. Although there was the suggestion of urinary sediment during admission (Table 2), this was presumed to be due to trauma from Foley insertion and possible superimposed bacterial colonization at that time. Given the patient’s negative serology and improving renal indices with fluid management, further autoimmune workup with renal biopsy was held during his hospitalization with the plan to follow up on the resolution of his kidney injury as an outpatient.

**Figure 3:**
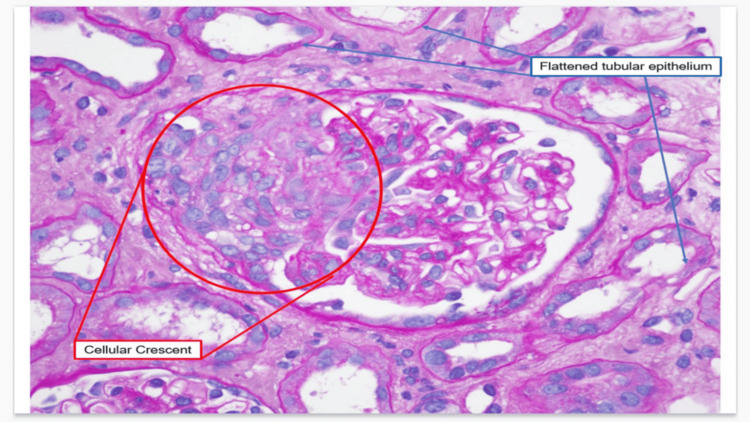
Renal biopsy demonstrating pauci-immune glomerulonephritis Hematoxylin and eosin (H&E) stained section of renal tissue showing a glomerulus with a cellular crescent (red circle) and adjacent tubules lined by flattened epithelium (blue arrows), indicating acute tubular injury. These findings are characteristic of pauci-immune crescentic glomerulonephritis, supporting the diagnosis of microscopic polyangiitis in the absence of detectable ANCA. ANCA, anti-neutrophil cytoplasmic antibodies

During his follow-up, there was a plateau in creatinine resolution. However, persistent hematuria and worsening proteinuria (protein-to-creatinine ratio rising to 926 mg/g and one month later to 1.7 g/g [Table 2]) led to a renal biopsy (Figure 3), performed via ultrasound-guided percutaneous approach.

The light microscopy showed 10 glomeruli, two of which had cellular crescents. The mesangium was unremarkable. Immunofluorescence stained negative for IgG, IgM, IgA, C3, C4, kappa, and lambda. Electron microscopy revealed ischemic glomeruli with moderate effacement of foot processes and no electron-dense deposits (Table 4). A diagnosis of pauci-immune glomerulonephritis with focal concentric features, consistent with MPA, was made.

**Table 4:** Histopathologic and Immunostaining Results from Jejunal and Renal Biopsies The jejunal biopsy demonstrated active enteritis characterized by ulceration and fibroinflammatory debris, with no evidence of malignancy. Immunohistochemical staining for CMV was negative, ruling out viral infection as the cause. These findings are consistent with vasculitic involvement of the gastrointestinal tract in microscopic polyangiitis. The renal biopsy revealed pauci-immune glomerulonephritis, with 2 out of 10 glomeruli showing cellular crescents on light microscopy. The mesangium appeared normal, and there was mild interstitial inflammation with acute tubular injury. Immunofluorescence studies were negative for immunoglobulin and complement deposition (IgG, IgM, IgA, C3, C4, kappa, and lambda), confirming the pauci-immune nature of glomerulonephritis. Electron microscopy showed moderate (40-50%) foot process effacement without electron-dense deposits, further supporting the diagnosis of pauci-immune disease. Together, these findings establish the diagnosis of ANCA-negative microscopic polyangiitis affecting both the kidneys and gastrointestinal system. The absence of immune complex deposition, negative viral studies, and lack of malignant features help exclude alternative diagnoses. The concordance between the renal and gastrointestinal pathology underscores the systemic nature of this vasculitis. CMV, cytomegalovirus

Test/Analysis	Results	Comments
Distal jejunum biopsy	Active enteritis with ulcer and fibroinflammatory debris	No evidence of malignancy
Jejunal mass biopsy	Active enteritis with ulcer and fibroinflammatory debris	No evidence of malignancy
Immunohistochemical stain (CMV)	Negative	No CMV identified
Renal biopsy	10 glomeruli, two with active cellular crescents; normal capillary loops, unremarkable mesangium	Mild interstitial inflammation, degenerative tubular changes, mild arterial and arteriolar sclerosis.
Electron microscopy (renal)	Moderate foot process effacement (40-50%), no electron-dense deposits	Consistent with focal concentric glomerulonephritis (pauci-immune type).

The patient was initiated on oral prednisone 60 mg daily with plans to commence cyclophosphamide. However, he required readmission two weeks later for the management of necrotizing fasciitis of the left thigh, necessitating surgical debridement and intravenous antibiotics. During this hospitalization, his renal function normalized (creatinine 1.17 mg/dL from baseline 1.3 mg/dL), with resolution of proteinuria (0.19 g/g). Repeat enteroscopy demonstrated complete healing of the jejunal lesions. Given this infectious complication coupled with apparent disease quiescence, the multidisciplinary team elected for close surveillance over immediate immunosuppression, with plans to initiate rituximab should renal or GI manifestations recur.

## Discussion

This case highlights the complexities in diagnosing MPA, particularly in patients with atypical GI and renal symptoms. While MPA is a rare form of ANCA-associated vasculitis, typically affecting the kidneys and lungs [1], it can infrequently present with GI symptoms such as abdominal pain, vomiting, and enteritis. Endoscopy and biopsy confirmed enteritis with ulceration in the jejunum, and CT chest findings of nodular bibasilar infiltrates and small pleural effusions pointed to probable systemic inflammatory involvement [2].

Although robust studies have linked ANCA-induced neutrophil activation to MPA, seronegative cases, such as this one, underscores the necessity of histopathological confirmation [3]. In this case, the diagnosis was confirmed through renal biopsy, revealing pauci-immune glomerulonephritis with cellular crescents. This emphasizes the importance of histopathological evaluation when serological testing is inconclusive [4].

GI involvement in this patient was notable, as such symptoms are not a predominant feature highlighted in the literature on MPA [5]. Gastroenterology consultation was pivotal in guiding the diagnosis. MPA itself presenting with GI symptoms is not particularly rare, but endoscopic evidence is uncommon [6]. The combination of GI symptoms and pulmonary-renal dysfunction prompted further investigation, leading to a diagnosis that may have been missed if only typical manifestations were considered. This case underscores the value of a multidisciplinary approach, with nephrology, rheumatology, and gastroenterology collaborating for diagnosis and management [7].

ANCA-negative MPA has sparked debate about the pathogenic role of PR3- and MPO-ANCAs as the sole inciting pathophysiologic process. While certain subsets of ANCAs, particularly those directed against specific epitopes, may be pathogenic and correlate with disease activity, routine testing may fail to detect these relevant antibodies, as seen in this case [8]. Studies in mice suggest that antibodies to hLAMP-2, a protein on neutrophils and endothelial cells, can activate neutrophils and cause kidney damage. The cross-reactivity between hLAMP-2 and bacterial proteins such as FimH, which is expressed in gram-negative bacteria, suggests that gram-negative bacteria could trigger pauci-immune vasculitis, potentially explaining this patient’s GI symptoms [9].

Treatment for pauci-immune MPA, whether ANCA-positive or ANCA-negative, typically involves high-dose corticosteroids and immunosuppressive agents such as cyclophosphamide or rituximab, especially in severe cases. The American College of Rheumatology (ACR) and the Vasculitis Foundation recommend these agents for the induction and maintenance of remission in MPA [10]. The Kidney Disease: Improving Global Outcomes (KDIGO) guidelines also support this approach with structured glucocorticoid tapering.

This case underscores the importance of a multidisciplinary approach with nephrology, pulmonology, rheumatology, and gastroenterology in diagnosing and subsequently managing MPA particularly when GI involvement is present. This case also highlights the challenges of managing ANCA-negative MPA in the setting of severe infection. While induction immunosuppression with cyclophosphamide was initially planned, the development of life-threatening necrotizing fasciitis necessitated treatment deferral. This underscores the importance of individualized risk-benefit assessment, particularly in seronegative cases where disease activity may be harder to monitor. Shared decision-making with all specialists involved became paramount in this clinical scenario.

## Conclusions

This case highlights that MPA, though typically associated with renal and pulmonary involvement, can present with rare GI symptoms. The negative ANCA result underscores the importance of histological evaluation in diagnosing pauci-immune glomerulonephritis. Early recognition of GI involvement and timely renal biopsy are crucial for accurate diagnosis and treatment initiation.

Clinicians should maintain a low threshold to suspect vasculitis in patients with multisystem dysfunction, even when ANCA testing is negative, as MPA can manifest with subtle, atypical features such as enteritis. A multidisciplinary approach, integrating clinical, histopathological, and radiological findings, is essential for effective management. This case reinforces the need for heightened awareness of atypical presentations to ensure prompt intervention and better outcomes.

## Appendices

Appendix A 

**Table 5:** Gastrointestinal pathogen panel (multiplex PCR) targets The GI PCR panel detects nucleic acids of the listed pathogens. Testing was performed per manufacturer protocols at Overlook Medical Center clinical microbiology laboratory. All targets were negative in this case.

Pathogen Category	Specific Targets Tested
Bacteria	*Salmonella* spp., *Shigella* spp., *Campylobacter* spp., *Yersinia enterocolitica*, Shiga toxin-producing *E. coli *(STEC), Enterotoxigenic *E. coli* (ETEC), *Vibrio *spp., *Clostridioides* *difficile* toxin B
Viruses	Norovirus GI/GII, Rotavirus A, Adenovirus F40/41, Astrovirus, Sapovirus
Parasites	*Giardia lamblia*, *Cryptosporidium* spp., *Entamoeba histolytica*, *Cyclospora cayetanensis*
Additional targets	Enteroaggregative *E. coli* (EAEC), Enteropathogenic *E. coli *(EPEC), Enteroinvasive *E. coli* (EIEC)
